# Early Outcomes of a Hip Arthroplasty Program in Rural Ethiopia

**DOI:** 10.2106/JBJS.OA.26.00114

**Published:** 2026-06-15

**Authors:** Gutu Daba Kumsa, Dawit Yosef Barkesa, Abduselam Muhamed Abda, Carlos Williams, Asana Bersisa Mirkena, Gavin Button

**Affiliations:** 1 Negele Arsi General Hospital and Medical College, Negele Arsi, Ethiopia

## Abstract

**Background:**

Hip arthroplasty profoundly improves quality of life. This procedure is primarily performed in the developed world by specialist surgeons and staff using sophisticated implants. Only rarely, in a few large cities, is hip arthroplasty performed in Africa. The objective of this study was to assess whether a de novo hip arthroplasty program in rural Ethiopia, at Negele Arsi General Hospital, is feasible.

**Methods:**

This cohort study analyzed data from 132 hip replacements in a total of 116 patients, the number and type of complications, and change in hip disability and osteoarthritis outcome scores (HOOSs). Preoperative and postoperative HOOSs were compared for 68 surgeries using a paired t-test; 58 surgeries had only postoperative HOOS data.

**Results:**

Between September 2021 and March 2025, a total of 97 primary total hip arthroplasty (THA) and 35 hemiarthroplasty (HA) procedures were performed, 29 by an Ethiopian team alone. The mean age of THA patients was 51 years, and 26% of patients were female. The primary indication was avascular necrosis for 41% of patients and primary osteoarthritis for 38% of patients. There was a statistically significant increase in mean HOOS subscore for THA patients from 46 to 85 and in HA from 31 to 87. There was an 11.4% complication rate with 4 failed acetabular shells, 3 femoral stem fractures, 3 dislocations, 1 deep venous thrombosis, 1 death, and no infections.

**Conclusions:**

It is possible to develop a hip arthroplasty program in a rural city in a developing country with outcomes that substantially improve patient quality of life. Early results of this study are promising; however, long-term follow-up and outcomes of Ethiopian-led surgeries alone are required to fully assess program success.

**Level of Evidence:**

Level III. See Instructions for Authors for a complete description of levels of evidence

## Introduction

Hip arthroplasty has been named the surgery of the century due to its profound impact on quality of life. Approximately 581,000 primary total hip arthroplasties (THAs) were performed in the United States in 2022^1^. Despite the high prevalence in the United States and the industrialized world, THA is infrequently performed in the developing world. The number of THAs performed in Africa is not known because of the lack of joint registries and reliable data reporting. However, a 2019 meta-analysis of all reports from sub-Sahara Africa (excluding South Africa) noted just 606 THA cases from 2009 through 2018.^2^

Malawi developed a joint registry in 2005, and reports have been published on up to 102 patients^3^. These reports have been observational, over multiple hospitals, and with limited outcome data. Other limited reports have come out of Ghana, Burkina Faso, Benin, Kenya, Egypt, Nigeria, and Botswana^4-10^. They have generally been singular reports based in large city hospitals.

Ethiopia, with 132 million people, is the second-most populous nation in Africa. Before the work described in this article, only 9 private hospitals in the capital and 1 missionary hospital outside the capital provided THA. One center, CURE hospital in Addis, had reported on 50 THAs performed from 2009 to 2013^11^, but it is no longer providing the service.

Barriers to care in Ethiopia and the developing world are many. They include the high cost of implants, lack of implants within the countries, challenges in importing goods, lack of surgeon education, lack of nurses and physiotherapists trained in arthroplasty, and lack of infrastructure, including a regular supply of electricity and running water^11^. Total hip implants are not sold in Ethiopia, and imported goods are highly restricted and taxed. There is also a lack of exposure to hip arthroplasty throughout the country, even among residents graduating from an orthopaedic residency. The first orthopaedic residency program was created at Black Lion Hospital in 1987. As of 2022, a total of 260 graduates had completed residency^12^. Recent graduates, including an author of this report (A.B.M.), did no more than assist on a handful of THAs during their training.

Development of a hip arthroplasty at Negele Arsi General Hospital (NAGH) began in 2019, when an American orthopaedic surgeon (G.B.) traveled to NAGH for a visit to assess feasibility. Subsequently, RCH Orthopaedics, a reliable provider of orthopaedic implants, was identified from India. RCH was chosen since all US-based implant companies that were approached for implant donations retrenched during the COVID pandemic and none could provide a long-term supply of cost-effective implants. Initial implant purchases were financed through individual donations since grant applications for a new project were unsuccessful. Older power equipment, helmets, and specialized surgical equipment that were being taken out of service in the United States were and continue to be donated by a variety of manufacturers.

In 2021, the consulting surgeon returned with a physical therapy assistant, scrub technician, and the necessary equipment (Coban, surgical hoods, stockinettes, Ioban, and power equipment) to perform THA safely. The local team had designed a lateral positioning device. Implants and instruments were previously delivered, including 125 femoral stems, 88 acetabular components and liners, and 52 bipolar heads. Over time, trochanteric plates and cables were obtained and a full supply of osteotomes and curettes were acquired to treat possible complications, in addition to long revision stems. Lectures and protocols were provided regarding patient selection, preoperative and postoperative care, and surgical technique. Owing to a lack of orthopaedic graduates in the country, hip arthroplasty was originally taught to a general surgeon who had never previously witnessed it.

The surgical team returned 11 times over the next 4.5 years. A resupply and update of implants occurred 8 times over this period. A second consulting orthopaedic surgeon (C.W.), fellowship-trained in adult reconstruction, participated in 3 of these missions, and a physical therapist and scrub technician participated at least 5 times each. By the conclusion of 5 missions, the operating room nursing team and physical therapy team had been adequately educated. At the conclusion of the 12th surgical mission and approximately 100 hip surgeries, the local orthopedist who had been hired (A.B.M.) was cleared by the original consulting surgeon to independently perform THA. He and the general surgeon did hemiarthroplasties after 3 missions.

The goal of the original 3 missions was to teach hemiarthroplasty (HA) so that patients with a femoral neck fracture might receive care. With subsequent missions, the goal became to train local surgeons to independently perform THA as well. The objective of the current study was to assess feasibility and early outcomes of the hip arthroplasty program. To assess change in health-related quality of life, preoperative and postoperative hip disability and osteoarthritis outcome scores (HOOSs) were collected in 51 THA and 17 HA procedures as well as postoperative HOOS for 58 procedures. Data on all complications were collected. Follow-up HOOS data are now collected on an annual basis. We hypothesized that there would be a statistically significant increase in HOOS that surpassed a minimal clinically important difference and that complication rates would be comparable with data reported from other African studies based in large cities and performed by specialists.

## Methods

### Study Design

In this cohort study, we analyzed data for all 116 patients and 132 index procedures of the NAGH hip arthroplasty program from September 2021 through March 2025. We assessed the number and type of complications and change in HOOS.

### Setting

NAGH is 4 hours by direct car from the capital of Ethiopia, Addis Ababa.

### Patient Selection

All skeletally mature patients presenting with significant arthritic disease were considered for surgery. The exclusion criteria were active infection, medical comorbidities too great for the surgical procedure, and radiographic joint destruction and/or limb shortening to a point where a primary press fit stem and standard acetabular shell would likely not succeed. Patients were preselected via radiographic review from the United States. The local internal medicine and anesthesia teams prescreened patients with a thorough laboratory investigation, electrocardiography, and echocardiogram in those older than 50.

### Surgical Technique

A posterior approach was used in all cases. Clindamycin IV was administered perioperatively due to a lack of cefazolin in Ethiopia and continued for another 5 doses postoperatively due to the potentially increased risk of infection. A dilute betadine soak was performed for 3 minutes before closure, and 1 g vancomycin powder was introduced before deep closure. After 8 missions, IV tranexamic acid administration and betadine nose swabs were used. All patients received a liner with 4 mm of lateralization and a 10° rim elevation to minimize the risk of dislocation, especially in the setting of femoral stems that each only had 1 offset. A decision was made to use a press-fit femoral stem due to the lack of bone cement in Ethiopia, challenges with importing cement, a lack of mixing devices, and the young age of most patients. In addition, temperature control in the operating room remains challenging, with no central air conditioning and a warm climate. A limited number of cemented procedures have now been performed in cases of significant osteoporosis.

### Postoperative Management

Abduction pillows were used for 48 hours. 81 mg aspirin was used twice daily for deep venous thrombosis prophylaxis except in a few higher-risk patients in whom subcutaneous unfractionated heparin was used. All patients were mobilized on postoperative day 0 or 1 and twice daily after that. A dedicated nursing team was educated to become hip physiotherapists. Postoperative hip exercise handouts were provided to all patients with illustrations and later were translated into the 2 main local languages (Afaan Oromo and Amharic). Education regarding posterior hip precautions was provided with insistence that patients avoid excessively squatting to use the bathroom (which is typical in Ethiopia) nor sleep on a mattress on the floor.

### Outcome Assessments

Starting in the ninth mission (May 2024), preoperative and 3 month postoperative HOOS were obtained on all surgeries and efforts were made to collect postoperative HOOS from prior patients. Postoperative only HOOS data ranged from 9 to 36 months. HOOS measures hip-related symptoms and function using a 40-item questionnaire to rate pain, symptoms and stiffness, activities of daily living, function in sports and recreation, and quality of life. Data are standardized so that a score of 0 equates to extreme disability and 100 represents no pain or limitations.

### Statistical Analysis

A paired *t* test, using SPSS-27 software, was used to look for statistically significant differences between HOOS subscores in patients with preoperative and postoperative data. Assumptions of normality were tested by the Shapiro-Wilk test. Effect size was analyzed using Cohen d.

### Ethics Clearance and Funding

The NAGH ethics committee authorized this research. No outside funding was provided.

## Results

**Fig. 1:**
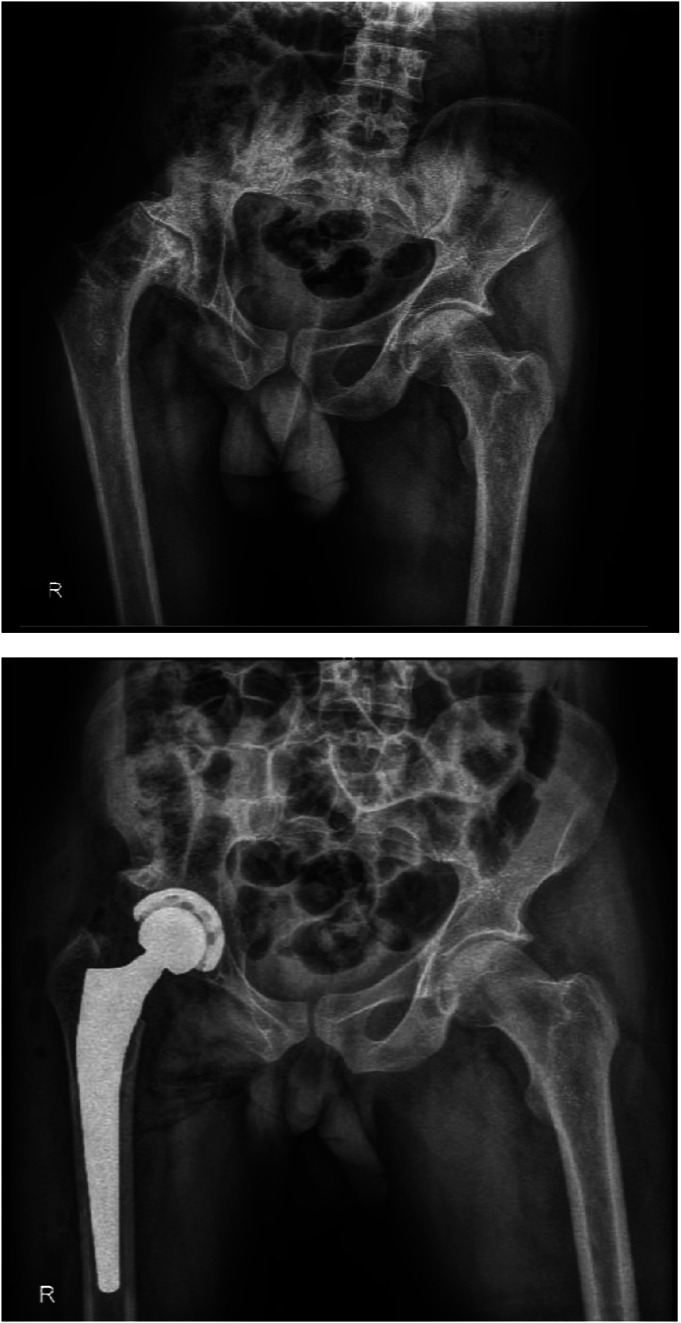
Preoperative and postoperative radiographs of a 28-year-old male patient with avascular necrosis.

A total of 97 THA and 35 HA procedures were performed, not including revisions. Eighty three THA and 20 HA procedures were performed by US-led missions. Fourteen THA and 15 HA procedures were performed by Ethiopian surgeons alone. Sixteen patients had bilateral THA. The mean age was 51 for THA patients and 65 for HA patients (Table I), and 26% of patients were female. All patients self-identified as Ethiopian, with the exception of 1 patient from Somalia and 1 from Kenya. The most common preoperative diagnosis for THA was avascular necrosis (41%), and the second-most common indication was osteoarthritis (38%; Table II). All HA cases were performed for a femoral neck fracture. Four patients were human immunodeficiency virus-positive, 2 had HA, and 2 had THA. Only 3 patients had prior tuberculosis of the hip all of which were in the US-led THA patient cohort. No patients had sickle-cell disease, and only 1 patient with preoperative anemia due to chronic kidney disease had postoperative anemia. No patients required a blood transfusion. Three patients had prior hip surgery with failed fracture fixation before the index THA. Five patients had failed core decompression surgery before a THA. Thirty percent of patients underwent general anesthesia, and 70% underwent spinal anesthesia. Owing to a very high prevalence of Muslim and Orthodox Christian patients, no patients were identified who smoked nor consumed alcohol regularly. Fig. 1 demonstrates a 28-year-old man with avascular necrosis.

**Table I:** Age Distribution of 81 THA Patients

Age Range	Total Number Patients (%)	US-Led Mission Total Patients	Ethiopian-Led Mission Total Patients
20–29	5 (6.2%)	4	1
30–39	11 (13.6%)	10	1
40–49	20 (24.7%)	16	4
50–59	23 (28.4%)	23	0
60–69	17 (21.0%)	13	4
70–79	3 (3.7%)	2	1
80–89	2 (2.5%)	2	0

**Table II:** Surgical Indications for Index THA in 97 Procedures

Indication	Number of Patients
Avascular necrosis	47
Osteoarthritis	33
Neglected femoral neck fracture	10
Posttuberculosis arthritis	3
Failed prior surgery for fracture	2
Posttraumatic arthritis	2

### Complications

Complications were recorded from program inception in September 2021 and encompassed a follow-up ranging from 3 to 44 months for US-led cases and 3 to 33 months for Ethiopian-led procedures. There were 15 complications in 81 THA patients (97 THAs) and no complications in 35 HA patients. Eight of the complications were due to implant design. Four patients required acetabular component revision for aseptic loosening. Two patients fractured their femoral stems, and both had the smallest stems. One of them subsequently fractured a stem 1 size larger. Broken stems were removed via bone windows distal to the fracture. One patient required femoral head exchange due to a manufacturing defect in a batch of femoral stems, resulting in a femoral head that did not seat firmly on the stem. One patient underwent a Girdlestone resection before multihole shells were available, when the acetabular component failed due to deficient acetabular bone stock. One patient had femoral component revision due to misplacement. Three patients had dislocations all of which were closed reduced without recurrence. There was 1 perioperative mortality on postoperative day 3 due to presumed myocardial infarction. There was 1 deep venous thrombosis and no known pulmonary emboli. There were no known superficial or deep infections. US-led missions had 11 complications in 83 THA and 20 HA, with implant failure (7) being the predominant complication, along with 3 dislocations. Ethiopian-led surgeries had 1 death and no other complications in 14 THA and 15 HA. No Ethiopian-led surgeries required revision.

### HOOSs

**Fig. 2:**
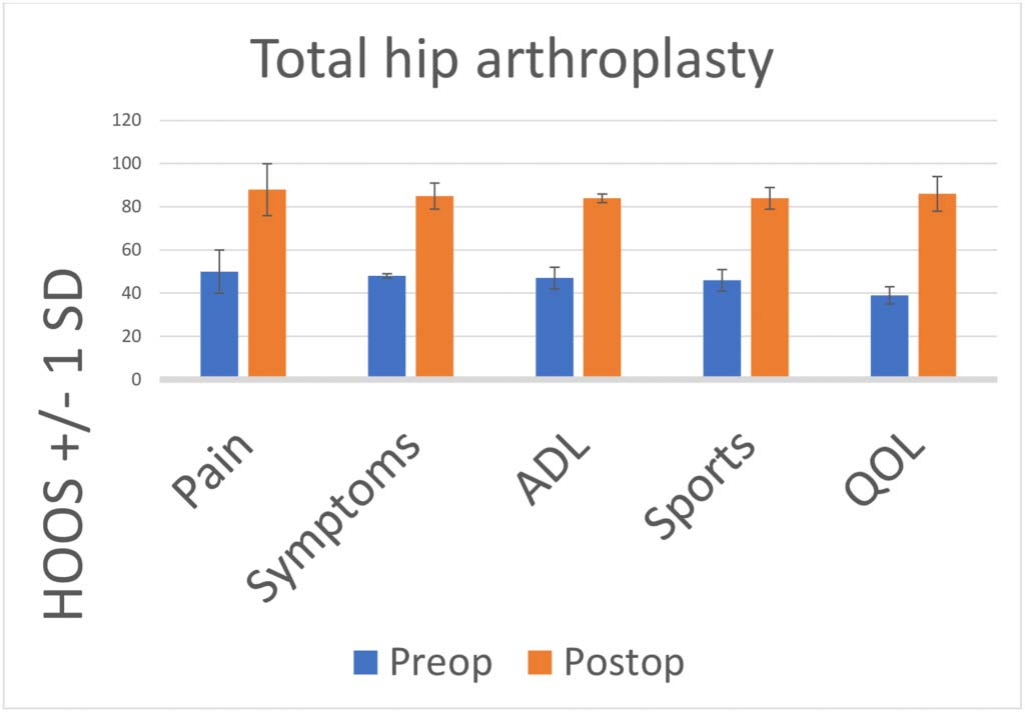
This represents the change in mean HOOS subscores±1 standard deviation from preoperatively to 3 months postoperatively in 40 total hip patients. HOOS subscores were pain, symptoms, activities of daily living, sports and recreation, and quality of life. HOOS = hip disability and osteoarthritis outcome score.

**Fig. 3:**
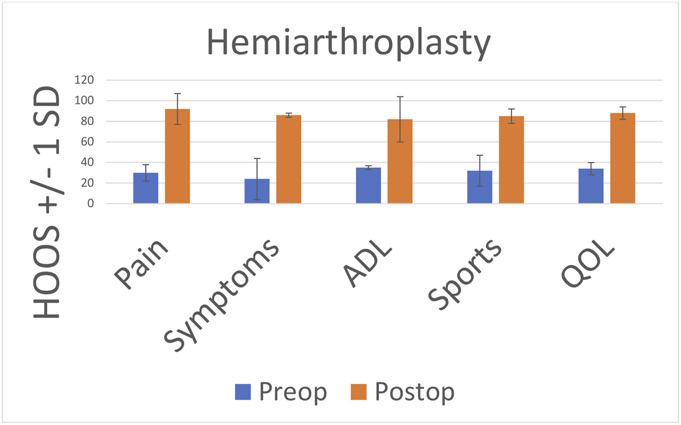
This represents the change in mean HOOS subscores ± 1 standard deviation from preoperatively to 3 months postoperatively in 17 HA patients. HOOS subscores were pain, symptoms, activities of daily living, sports and recreation, and quality of life. HA = hemiarthroplasty, and HOOS = hip disability and osteoarthritis outcome score.

Preoperative and 3-month postoperative HOOSs were obtained for 40 patients who underwent 51 THAs (11 bilateral) and 17 HA patients. There was a statistically significant increase in mean HOOS score for THA patients from 46 to 85 (p < 0.001, mean difference 39, 95% CI 35-45) and in HA from 31 to 87 (p < 0.001, mean difference 55, 95% CI 51-59). All subscores increased by at least 37 points in every category in both cohorts. The increases were additionally statistically significant for every HOOS subscore, for both THA and HA patients (all p < 0.001), and well surpass the generally accepted minimal clinically important difference of 18 on the HOOS scale^13^ (Figs 2 and 3 and Supplementary Table I). Normality, which was tested for all data pairs, was normal. Effect size ranged from 1.9 to 8.8 when comparing mean HOOS subscores, indicating a large effect.

HOOS scores in US-led THA surgeries increased from 46 to 85 (p < 0.001, mean difference 40, 95% CI 35-45). HOOS scores in Ethiopian-led THA surgeries increased from 50 to 88 (p < 0.001, mean difference 39, 95% CI 27-51). No THA patients were lost to follow-up. HOOS scores in US-led HA surgeries increased from 31 to 86 (p < 0.001, mean difference 55, 95% CI 50-60). HOOS scores in Ethiopian-led HA surgeries increased from 32 to 86 (p < 0.001, mean difference 54, 95% CI 48-60). Six patients with HA by the Ethiopian team were lost to follow-up, while no patients from US-led HA procedures were lost to follow-up.

An additional 41 patients with 46 THAs and a separate 12 HA patients had only postoperative HOOS collected. HOOS subscore mean averages for the 41 THA patients were pain, 90; symptoms, 88; activities of daily living, 89; sports, 85; and quality of life, 87. HA mean averages for 12 patients were pain, 81; symptoms, 88; activities of daily living, 83; sports, 63; and quality of life, 70. Follow-up HOOS data for this cohort ranged from 9 to 36 months.

## Discussion

HOOS outcome and complication data demonstrated that quality of life was significantly improved by the hip arthroplasty program at NAGH. HOOS subscores all increased at least 37 points. An 11.4% complication rate compares similarly with data from other sub-Saharan studies, with reported rates ranging from 6.9%^3^ to 26.1%^14^. These studies encompassed outcome data that ranged from 1^9^ to 5 years^15^. The 4 proven acetabular component in-growth failures were likely due to limited surface friction and porosity. Newer designed implants have been purchased, hopefully avoiding such future complications. Aseptic loosening has also been reported in multiple other African studies.^5,16,17^

No prior studies from sub-Saharan Africa have reported on HOOS, and only 5 studies^3,9,11,15,18^ have previously reported Harris Hip Score or Oxford Hip Score with both preoperative and postoperative data, none on as many as the 68 cases presented here. Only 3 other studies have outlined the development of a continuing arthroplasty program.^5,19,20^

Most challenges experienced in Ethiopia were noted elsewhere in Africa. At NAGH, the greatest challenge to establishing the program was the importing of goods. No implants are produced in Ethiopia, nor the African continent except South Africa, and strict rules exist regarding importing any goods. A similar challenge was noted in the Democratic Republic of Congo^20^. It took 1 year of work with a local customs agent for the first shipment of implants to be authorized to enter Ethiopia, now only 2 months are required for authorization. Establishment of stringent sterile technique was also challenging; the local operating room team previously reused materials such as bovie and suction tubing after cleaning with alcohol. Postoperative rehabilitation was only successful in Ethiopia due to attendance of a physical therapist for the first 5 missions and education of local nurses in hip physiotherapy. This was also noted in Botswana and Burkina Faso.^5,19^

From a surgical standpoint, the greatest challenge was the very late disease presentation seen in Africa. Other authors^5^ have noted this challenge as well, with many patients presenting with a leg length discrepancy over 5 cm or complete erosion of the acetabulum. With only a primary press-fit stem (for the first 8 missions) and an acetabular shell of only fair quality (first 10 missions), patient selection was critical. Patients with greater than a 5 cm leg-length discrepancy were excluded due to risk of neurologic injury and the inability to shorten the femur with a primary, press-fit stem. A stainless-steel stem was initially chosen to save on cost. The patients with stem fractures had the smallest stem and were men over 80 kg. Since those fractures, the 2 smallest stems are now titanium.

There were no superficial or deep infections, likely attributable to the attempt to reproduce US conditions (i.e.: Ioban, Coban, and sterile surgical hoods), stringent education of the operating room team by the scrub technician and surgeons over many missions, general good health and young age of the patients, good fortune, and short-term follow-up. The lack of infections should be interpreted with caution given the short-term follow-up. Although follow-up is challenging when patients may travel days to the hospital, it is probable that most complications would be reported. NAGH is one of only 10 hospitals performing THA in Ethiopia, and the senior Ethiopian author (A.B.M.) provides his cellular phone number to all patients.

### Financial Viability

Financial viability is likely now even without significant US financial support. After the first surgical mission, patients were charged approximately $1,200 to cover implant ($750) and labor ($450) costs. All costs are now covered by patient fees.

### Program Development

Development of a similar program would be primarily dependent on 1 or 2 surgeons willing to make multiple annual mission trips and take personal ownership of a program. Local surgeons do not have the exposure to surgical technique nor the knowledge of infrastructure necessary to successfully perform joint arthroplasty de novo. Once such surgeons have been identified the next critical step is to identify a source of quality implants and obtain help importing such goods. Nursing and surgeon education and careful, step-wise patient selection are the subsequent steps that are required. Finally, success was also dependent on strong local hospital leadership. Patient recruitment, staff training, and program growth and improvement that is required for success occurred because of strong leadership both at the CEO and local surgeon levels.

### Strengths

No studies of this size with preoperative and postoperative outcome data have been previously reported from sub-Saharan Africa. There have been no prior reports from Africa describing the development of a hip arthroplasty program outside of large metropolitan areas. No prior studies have reported a hip replacement program continued by local surgeons when missionaries are not present.

### Limitations

Outcomes have been very short for joint arthroplasty, ranging from 3 months to nearly 4 years, and preoperative and postoperative data are available for only 68 procedures with just 3 month HOOS postoperative data. Longer-term follow-up is required to assess the performance of the program. Nearly half the data were collected only retrospectively and observer bias exists as it was collected by members of the hospital team by phone interview. It is also impossible to yet assess the success of the training until there are a larger number of cases performed only by local surgeons who tackle more challenging cases. Although complication rates were lower in Ethiopian-led surgeries (1 death, no other complications), that is likely mainly due to Ethiopian THA cases being self-selected as simpler with less dysplasia and leg length inequality to maximize outcomes, while more challenging cases were reserved for American-led missions. In addition, there were only 15 THAs in the Ethiopian-led group. Finally, 6 HA patients from Ethiopian-led missions were lost to follow-up (20% of all Ethiopian cases) and no HA patients from US-led missions, a source of possible bias when comparing group outcomes. The success of Ethiopian-led surgeries must still be demonstrated with long-term follow-up and more challenging surgical cases.

## Conclusions

Itis possible to develop a hip arthroplasty program in a rural city in a developing country with outcomes that substantially improve patient quality of life. Early results of this study are promising. However, long-term follow-up and outcomes of Ethiopian-led surgeries alone are required to judge success. For a program to be sustainable, it is mandatory to educate local orthopaedic surgeons and support staff over many missions and have a reliable supply of quality implants.

## Appendix

Supporting material provided by the authors is posted with the online version of this article as a data supplement at jbjs.org (http://links.lww.com/JBJSOA/B193). This content was not copyedited or verified by JBJS.
